# ATP bioluminescence values are significantly different depending upon material surface properties of the sampling location in hospitals

**DOI:** 10.1186/s13104-015-1757-9

**Published:** 2015-12-21

**Authors:** Tomoko Shimoda, Rika Yano, Shinji Nakamura, Mitsutaka Yoshida, Junji Matsuo, Sadako Yoshimura, Hiroyuki Yamaguchi

**Affiliations:** Department of Fundamental Nursing, Faculty of Health Sciences, Hokkaido University, Sapporo, Japan; Laboratory of Morphology and Image Analysis, Biomedical Research Center, Juntendo University Graduate School of Medicine, Tokyo, Japan; Department of Medical Laboratory Science, Faculty of Health Sciences, Hokkaido University, Nishi-5 Kita-12 Jo, Kita-ku, Sapporo, Hokkaido 060-0812 Japan

**Keywords:** Material properties, Hospital cleanliness, ATP bioluminescence, Stamp agar, Culture, Scanning electron microscopy

## Abstract

**Background:**

Our previous study into assessing hospital cleanliness in Japan by two common methods, ATP bioluminescence and the stamp agar method, revealed considerable variability in the data of both methods (BMC Research Notes, 7: 121, 2014). To investigate the reason(s) for the variability, we reanalyzed the data (*n* = 752) from the point of view of the material surface properties of sampling sites.

**Methods:**

Data obtained from surfaces with unknown properties and different purposes such as floor were omitted, and the remaining data (*n* = 488) were used for this study. The material surface properties on sampling sites were divided into six categories: melamine coated (*n* = 216), vinyl chloride (*n* = 16), stainless steel (*n* = 144), wood (*n* = 63), and acrylonitrile-butadiene styrene resin coated (*n* = 48). The data between individual material properties were compared.

**Results:**

The ATP values of high-touch places were significantly different depending on the type of surface, but no significant difference in stamp values between material properties was seen, indicating that in contrast to stamp values, ATP-accumulation more depends on the physical properties of the material surface such as electronic charges or roughness. To confirm this, we assessed a degree of roughness on vinyl chloride material surface (disutilized floor samples actually used for each of the hospitals) by observation with scanning electron microscope (SEM). As a result, SEM observation similarly revealed considerable roughness on the materials, which may allow microbes to contaminate the materials without noticing it.

**Conclusion:**

Material properties must be considered when evaluating hospital cleanliness with ATP values, and provide a strong warning into evaluating hospital cleanliness.

## Background

In the last decade, much attention has been focused on hospital-acquired infections caused by microbe-contaminated hospital environments. Surfaces that are frequently touched by hands, such as doorknobs, sinks, lockers, and tables of inpatients, presumably provide the greatest risk for patients [1–3]. Consequently, improving hand hygiene and isolation practices are critically important to control hospital-acquired infections, and routine cleaning practices in hospitals has been responsible for a decrease in transmission of hospital-acquired infections [4–7]. Hospital cleanliness protocols, according to guidelines such as the US. Centers for Disease Control and Prevention, include appropriate monitoring of hospital environments with a warning that visual assessment of hospital cleanliness is not enough to control hospital-acquired infections [8–10]. For monitoring hospital cleanliness, both the adenosine triphosphate (ATP) bioluminescence (ATP method), which is an indicator of general organic contamination [11–15], and the standard stamp agar method (stamp method), which is an indicator of microbiological contamination [16–18], has been used worldwide.

We have previously assessed hospital cleanliness using the ATP and stamp methods: the surfaces of 752 sites in nurse and patient areas in three hospitals located in a central area of Sapporo, Japan were evaluated by both methods (each of the surfaces was sampled eight times in 2 months). The results revealed the presence of a wide range of organic contamination spread via hand touching, including microbial contamination [19]. However, both methods indicated considerable variability regardless of daily visual assessment and wiping, and positive surfaces were irregularly seen, suggesting that ongoing daily hospital cleanliness is insufficient, although the exact reason for the variability remains unclear [19].

Several previous studies have suggested that in dental or food sciences, material surface properties influence microbial contamination [20–22]. In the present study, we reanalyzed the data from our previous study (*n* = 752) from the point of view of the material surface properties of the sampling sites. We show that material surface properties must be considered when evaluating high-touch places with ATP values, and provide a strong warning into overestimating hospital cleanliness.

## Methods

### Data used for this study

The 752 surfaces within nurse [instillation preparation table (nurse station); routine worktable (nurse station); nurse wagon (mobile station in nurse area); doorknob (nurse station)] and patient areas [guardrail in corridor (public space); hospital entrance floor (public space); locker for hospital inpatients (room with multiple beds); overbed table (room with multiple beds); locker for hospital inpatient (room with private bed); overbed table for hospital inpatient (room with private bed); windowsill (room with private bed); windowsill (room with multiple beds)] in three hospitals [“A” hospital (>500 beds), “B” hospital (100–500 beds), and “C” hospital (<100 beds)] located in a central area of Sapporo, Japan, have previously been evaluated by the ATP and stamp methods [19]. The dataset from this publication was re-used for the current study. As mentioned in the previous report, ATP bioluminescence was measured using a 3M Clean-Trace ATP System (10 × 10 cm^2^; Sumitomo 3M Limited, Tokyo, Japan), and the values were expressed as bioluminescence relative light units (RLU) [19]. Wiping the area of each surface was performed with a cotton swab supplied with the system. Also, the commercial stamp agar assay was based on soybean casein digest (10 cm^2^ surface area; Nissui Pharmaceutical Co., Ltd., Tokyo, Japan) for monitoring environments for microorganism contamination; the agar plate was cultured for 5–7 days under aerobic conditions at 30 °C, colonies were counted and these data were estimated as colony-forming unit (CFU) per 10 cm^2^ [19].

### Classification into individual material properties

**Fig. 1:**
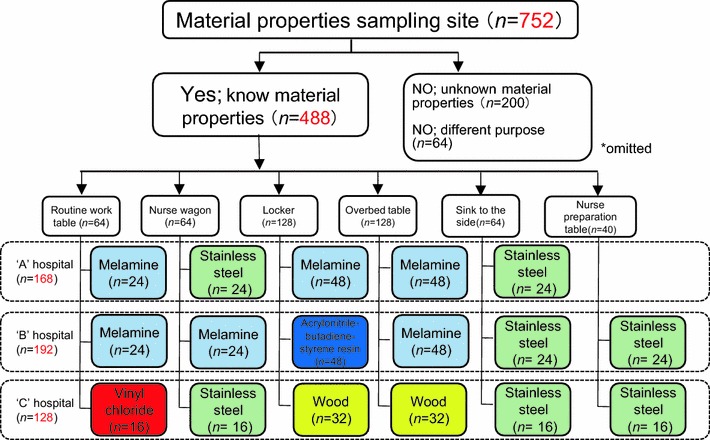
Classification of material surface properties. The material surface properties of the sampling sites were divided into six categories: melamine coated, vinyl chloride, stainless steel, wood, acrylonitrile-butadiene styrene resin coated, and antibacterial vinyl chloride. *because of one missing sample, there are 751 sites with ATP data

We contacted the manufacturers of various surfaces to determine the surface material properties. The data were divided into six categories: melamine coated (*n* = 216), vinyl chloride (*n* = 56), stainless steel (*n* = 144), wood (*n* = 63) and acrylonitrile-butadiene styrene resin coated (*n* = 48). Data obtained from unknown material properties (*n* = 200) and objects of different purpose (floor) (*n* = 64) were excluded, and the remaining data (*n* = 488) were used for this study. Fig. 1 summarizes the number of surfaces with each material per hospital.

### Ethical consideration

Since the study contained no human subjects or samples, the Faculty of Health Sciences at Hokkaido University or each hospital waived the need for ethical approval, including written consent [19]. Meanwhile, the study design was explained and consents for obtaining ATP or stamp samples into hospital environments were orally obtained from all medical staffs and hospital inpatients, which intend to participate in this study. Also, privacy and confidentiality of personal information was also protected, according to the Helsinki declaration [23].

### Scanning electron microscopy (SEM)

According to a previous method [24], floor material samples (approximately 1 × 1 cm) were washed in saline, fixed with 2.5 % (v/v) glutaraldehyde in phosphate-buffered saline (pH 7.4) for 2 h at room temperature, and subsequently soaked in 2 % (w/v) osmium tetroxide for 1 h at 4 °C. The samples were then dehydrated in ethanol, freeze-dried, and coated with osmium using a plasma osmium coater. Samples were analyzed using a Hitachi S-4800 SEM (Hitachi, Tokyo, Japan). The materials [disutilized floor samples actually used for each of the hospitals (A–C)] consisting of vinyl chloride (Hospital “B” floor material also contained antibacterial substance) were kindly provided by TOLI Corporation (Osaka, Japan) and TAJIMA ROOFINF (Tokyo, Japan).

### Statistical analysis

Comparison of the data between individual material properties was assessed by one-way analysis of variance and multiple regression analysis. A *p* value of <0.05 was considered statistically significant.

## Results

### ATP bioluminescence values, but not stamp values, differ significantly depending on material surface propertie*s*

**Fig. 2:**
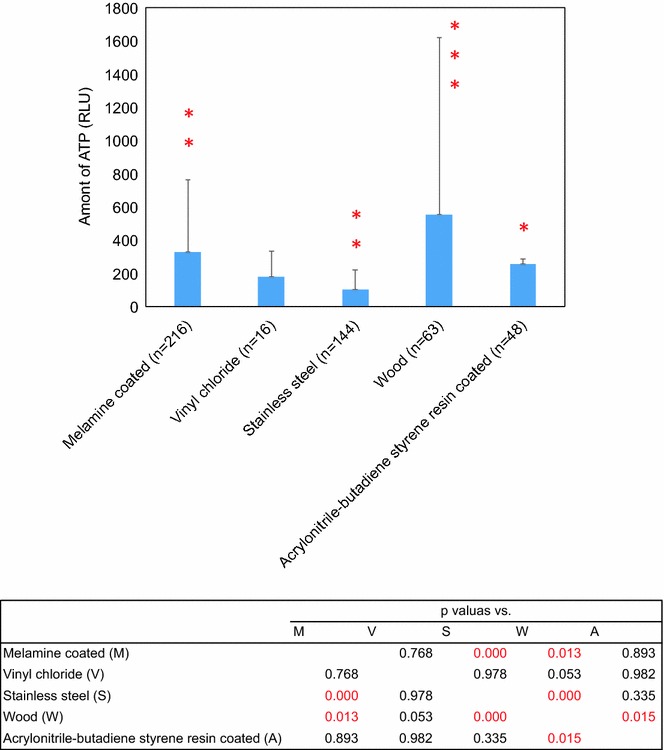
ATP amount estimated by the ATP method by each material. Values show the amount of ATP (RUL) +standard deviation/100 cm^2^. *p < 0.001 vs. the other class. *Asterisk* numbers also indicate the number of classes with a statistically significant difference. The values in the table show *p* values with statistical significant (*Red*)

The ATP values from high-touch places significantly differed in a material property-dependent manner (Fig. 2, *p* < 0.001). Each of the distinct material surfaces showed the following trends. Stainless steel showed the lowest ATP values among the categories, determining the surface as low-touch place, and acrylonitrile-butadiene styrene resin coated and vinyl chloride remained neutral, indicating medium-touch places. In contrast to these, both melamine coated surface and wood surface were relatively higher than the others, although wood surface were considerably in variable.

**Fig. 3:**
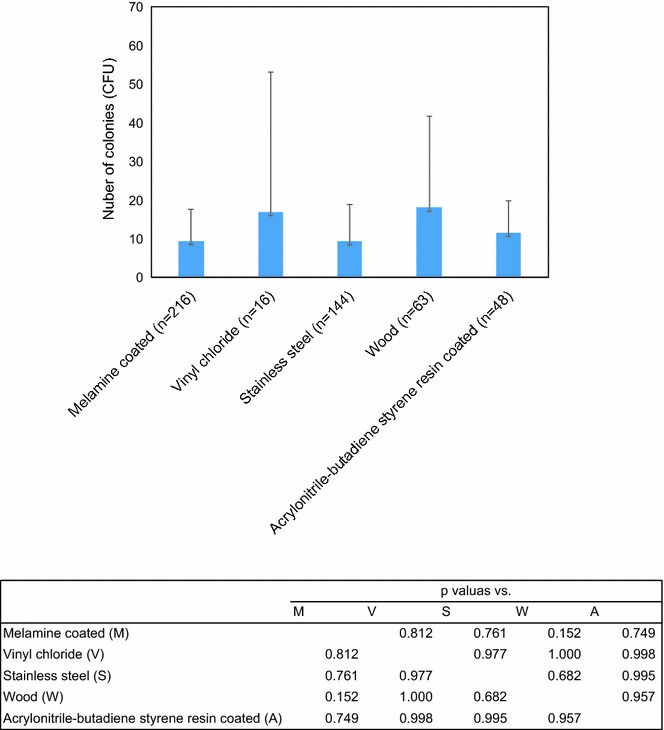
Comparison of CFU amount estimated by the stamp method by each material. Values show amount of CFU + standard deviation/10 cm^2^. The values in the table show *p* values

Meanwhile, the stamp values did not significantly differ by surface, although in contrast to ATP values stamp results regardless of material properties were considerably in variable (Fig. 3, *p* = 0.194).

Taken together, the findings indicated that in contrast to stamp values, ATP-accumulation more depends on the physical properties of the material surface such as electronic charges or roughness.

### Ultra-structures of vinyl chloride material surfaces (disutilized floor samples) shows considerable roughness

**Fig. 4:**
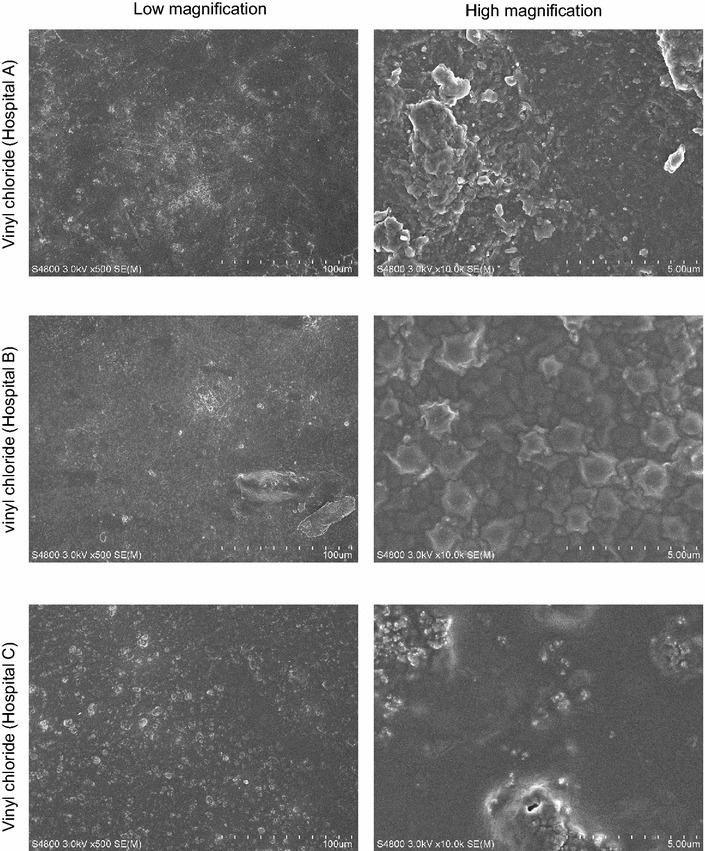
Representative SEM images showing ultra-structures of disutilized floor samples actually used for each of the hospital floors. *Scale bars* show 100 μm (low magnification) and 5 μm (high magnification), respectively. All samples consisting of vinyl chloride (‘Hospital B’ also with antibacterial materials) are disutilized floor materials. Samples ‘Hospital A, Hospital C’ and ‘Hospital B’ are provided by TOLI Corporation and TAJIMA ROOFINF, respectively

Vinyl chloride surfaces as well as melamine coated consisting of chemical polymers are believed to be very convenient materials, protecting from water leakage or pollution from dirty materials similar to stainless steel material. However, the data were contradictory to this notion, with moderate values of ATP test. To explore this contradiction, ultra-structures of vinyl chloride surface materials (disutilized floor samples actually used for each of the hospitals) were visualized by SEM. As a result, all the SEM images similarly revealed that there is extreme roughness on the vinyl chloride surfaces, which may allow space for colonizing microbes without noticing it (Fig. 4, high magnification). As expected, our data showed actual floor swab samples with moderate values of ATP test, indicating a dirty place (data not shown).

Thus, these data support that in contrast to stamp values, ATP-accumulation results more depend on the material surface physical properties such as roughness, suggesting that the ATP method may overestimate hospital cleanliness.

## Discussion

Stainless steel surfaces are believed to maintain cleanliness relatively well, with minimal attachment of organic constituents. However, our analysis revealed that even though stainless steel may be determined as clean by the ATP method, stamp values were not identical to this trend, indicating that microbial contamination does occur. Although the reason for this inconsistency is unknown, several studies have indicated that attachment of microbes on stainless steel surfaces is significantly influenced by temperature or humidity, and that stainless steel can support microbial survival [25]. Additionally, if given weeks to grow, microbes appear to have an inherent ability to colonize any stainless steel or polymeric material generally used for high-touch surfaces in hospitals [26]. Taken together with our data, we caution that ATP testing on stainless steel of hospital likely overestimates hospital cleanliness.

In contrast to stainless surface, ATP results on wood surfaces were relatively higher than those of the other materials even though considerably in variable, indicating that wood surfaces were more high-touched than vinyl chloride or stainless steel. However, the exact reason why ATP values on wood surfaces were considerably in variable remains unknown. Meanwhile, we found the fact that all wood material surfaces assessed into the study were coated with melamine polymer, although the coating amount or timing could not be found out. Therefore, it cannot deny that difference of the polymer coating degree to protect wood surface could influence the ATP evaluation. Thus, we also caution that ATP values on wood surface are likely to be inaccurate, and less wood material in hospitals may be useful to improve hospital cleanliness.

In general, materials consisting of vinyl chloride as well as melamine are chemical polymers and are commonly used in daily goods such as tables, bed frames, dishes, and floors. Such chemical polymers are believed to be very convenient materials, protecting from water leakage or pollution from dirty materials, which presumably makes their surfaces easier to keep free of microbial contamination. However, the data were contradictory to this notion, with moderate values of ATP test, implying the presence of unknown physical factor such as roughness with changes depending on wears, which provides microbes with hiding places. We therefore attempted to see a roughness degree of the material surface by the observation with SEM. As expected, the SEM images revealed an extreme roughness on the vinyl chloride surfaces, allowing space for colonizing microbes, easily altered by a degree of wears. In addition, it cannot deny that since ATP assessment is done by hard swab included in the commercial kit, sampling on rough surfaces may inefficiently collect organic material. Thus, although the data are limited, the observation provides us with a caution that cleanliness of surfaces containing chemical polymers with a distinct degree of roughness may be overestimated, and suggests a need for more adequate hospital cleanliness monitoring protocols.

The reason why ATP values were significantly different in a material property-dependent manner is currently unknown. Meanwhile, environmental ATP is sufficiently stable to ensure environmental cleaning in health care, and has been experimentally demonstrated that in contrast to liquid suspension, ATP on dried surfaces remained very stable for 29 h [27]. Taken together with this, our results suggest that while in daily monitoring assessments ATP deterioration is minimal [19], ATP accumulation may dramatically change in a material property-dependent manner. Thus, use of the ATP test alone may lead to an overestimation of hospital cleanliness when used for immediate feedback of cleaning coverage.

Benchmark values of ATP and stamp evaluation into assessing environmental cleanliness have been already proposed as 250 RLU/10 cm^2^ and 2.5–5 CFU/cm^2^, respectively [12, 28]. Meanwhile, as described previously [19], our system revealed that the detection limits of the ATP method (according to the spiked experiment with *Escherichia coli*, *Staphylococcus aureus*, and *Bacillus subtilis*) and stamp method [according to taking samples from student tables (*n* = 19) in a lecture room of our department during vacation] were estimated at 12.7 RLU/10 cm^2^ and 0.5 CFU/cm^2^, respectively. Thus, although our detection limits were estimated with accuracy, these appeared to be relatively low when compared with the previous benchmark values. Furthermore, although the exact reason why its contradiction occurred also remains unknown at this time, the recent observation assessing hospital cleanliness in Japan with an estimated benchmark values similar to our detection limits, supported our data to be accurate [29].

According to cleaning and hand hygiene protocols, hospital environmental surfaces are believed to maintain minimal microbial contamination, and clean surfaces contribute to reducing infectious diseases in hospitals. However, hospital-acquired infections continue to result in a significant loss of life and high cost (e.g., the United States healthcare system estimated $45 billion annually) [30]. In support of this, our previous data have revealed that ongoing daily hospital cleanliness is not sufficient in hospitals with a tendency toward long-term care in Japan [19]. Thus, hospital cleanliness must be reevaluated from the point of view of material surfaces for preventing hospital-acquired infections, to establish more accurate cleanliness protocols and accurate monitoring systems.

## Conclusion

Taken together, we conclude that material surface properties must be considered in evaluating high-touch places with ATP values, and provide a strong warning to the possibility of overestimating hospital cleanliness with the use of the ATP test alone.

## Abbreviations

ATP: adenosine triphosphate
SEM: scanning electron microscope
CFU: colony-forming units
